# Risk Factors of Household Transmission of Pandemic (H1N1) 2009 among Patients Treated with Antivirals: A Prospective Study at a Primary Clinic in Japan

**DOI:** 10.1371/journal.pone.0031519

**Published:** 2012-02-16

**Authors:** Nobuo Hirotsu, Koji Wada, Hitoshi Oshitani

**Affiliations:** 1 Hirotsu Clinic, Kawasaki, Japan; 2 Department of Public Health, Kitasato University School of Medicine, Sagamihara, Japan; 3 Department of Virology, Tohoku University Graduate School of Medicine, Sendai, Japan; University of Hong Kong, Hong Kong

## Abstract

**Background:**

Household transmission of influenza can affect the daily lives of patients and their families and be a trigger for community transmission, thus it is necessary to take precautions to prevent household transmission. We aimed to determine the risks of household transmission of pandemic (H1N1) 2009 influenza virus from an index patient who visited a primary clinic and was treated with antiviral drugs.

**Methods:**

We followed up all the patients who were diagnosed with influenza A by rapid diagnostic test with a questionnaire or interview from July 2009 to April 2010. Secondary cases were defined as patients visiting the clinic or other clinics and being positive for influenza A by rapid diagnostic test within 7 days of onset of an index patient. Logistic regression analysis was used to explore the association between household transmission and the studied variables.

**Results:**

We recruited 591 index patients and 1629 household contacts. The crude secondary attack rate was 7.3% [95% confidence interval (CI): 6.1–8.7]. Age of index patients (0–6 years old: odds ratio 2.56; 95% CI: 1.31–4.01; 7–12 years old: 2.44, 1.31–3.72; 30–39 years old 3.88; 2.09–5.21; 40 years old or more 2.76; 1.17–4.53) and number of household members with five or more (3.09, 2.11–4.07), medication started ≥48 hours from the onset of fever (2.38, 1.17–3.87) were significantly associated with household transmission.

**Conclusions:**

Household transmission was associated with index patients aged ≤12 years old and adults ≥30 years with children, with more than five persons in the household, and medication initiated ≥48 hours from the onset of fever among the population, in which, antiviral treatment was given to all patients. We need to warn patients at high risk of household transmission to take additional precautions.

## Introduction

The household of an influenza patient could be at high risk of infection [1]–[3]. The secondary attack rate (SAR) of pandemic (H1N1) 2009 in 2009 was 10–45% [1]–[7]. Household transmission could result in the burden of taking care of patients at home for several days and affect their daily lives, and an additional financial burden because parents cannot go to work [8], [9]. Household transmission could also be a trigger of community transmission. Understanding risk factors of household transmission is critical for minimizing the impact of influenza. A variety of risk factors have been identified, such as age of the index patient and number of household members [2]–[4], [6], [10], [11]. Household transmission can be reduced if additional precautions, such as maintaining distance between patients and healthy household members, are implemented in household.

In Japan, the case fatality rate of influenza was relatively low comparing with other countries [12], [13]. One of the reasons could be the accessibility of antiviral medications for patients at primary clinics because of universal health coverage [13], [14]. However, treatment with neuraminidase inhibitors zanamivir or oseltamivir, for patients who are infected with influenza virus is not conclusive for prevention of household transmission of influenza [15], [16], even though the mechanisms of neuraminidase inhibitors interfere with the release of progeny influenza virus from infected cells, and are effective for treatment and resolving viral shedding [16], [17]. There have been studies on household transmission of influenza in cases when not all the patients have taken antiviral drugs, however, there has been few studies on risk factors of household transmission when all patients have been prescribed antiviral medication. The aim of the this observational cohort study in Japan was to determine risks of household transmission of pandemic (H1N1) 2009 virus from index patients who visited a primary clinic and took an antiviral drug during the first wave of influenza in 2009.

## Materials and Methods

### Data collection

We implemented this prospective cohort study at a primary clinic in Kawasaki city, Kanagawa, Japan (population 1,410,000) from 22 July 2009 to 19 April 2010 when the first epidemic wave of influenza occurred in Japan after the identification of pandemic (H1N1) 2009 influenza virus in Mexico in April 2009 [18].

We obtained clinical information on prescribed antiviral medicine from the medical chart and disseminated a self-administered questionnaire to all the index patients or their families. A questionnaire comprised the number in the household (including the index patient), time from onset of fever of the index patient to initiating drug treatment and to onset of symptoms of household members who had secondary transmission of influenza A, and the existence of household members who were diagnosed with influenza A within 7 days of onset of the index patient. A questionnaire was returned to the clinic by FAX, or by mail, or submitting at the counter of the clinic. In the case of a questionnaire not being returned, we contacted the patient or patient family to ask them to send it back or to be interviewed.

### Definition of index patient

We defined an index patient as the first person that had influenza-like symptoms, which were fever (≥37.5°C) plus cough and/or sore throat in his/her household, and were identified with influenza A virus by a rapid diagnostic test. In patients who had influenza-like illness but who were negative by a rapid diagnostic test, and the time interval from onset of symptoms to visiting the clinic was <24 hours, we performed a rapid diagnostic test on the next day, and prescribed antiviral medication based on diagnosis by a clinician. Households that consisted of at least two persons were eligible for study participation.

### Definition of household transmission

We defined household transmission as a household member who visited the same clinic or other clinics, who was then diagnosed with influenza A by rapid diagnostic test within 7 days after the index patient started to develop influenza like symptoms.

### Drug administration

All index patients received treatment with oseltamivir or zanamivir twice daily for 5 days, with the dose based on their body weight, as soon as patients were diagnosed with influenza. The choice of antiviral drug was not randomized and took into consideration patients' preferences for either oral or inhalational administration. In Japan, there are concerns that oseltamivir administration in teenagers can cause psychological and neuropsychiatric side effects [19]. Thus, in this study, oseltamivir for teenagers were prescribed only when the index patient seemed not to be able to inhale zanamivir. We did not prescribe antiviral drugs for prophylaxis for healthy household individuals.

### Statistical analysis

SARs were calculated according to the number of persons who were determined as “household transmission” divided by the total number of enrolled household members excluding the total number of index patients. Pearson's χ^2^ test was used to compare categorical variables and the *t* test for continuous variables. Logistic regression analysis with a generalized estimating equation was used to determine the association between household transmission and the studied variables. We first examined the variables by univariate analysis, and then by multivariate analysis without factors not significant at P = 0.10 in univariate analysis. All analyses were performed using IBM SPSS Statistics 19. Since the incidence of household transmission was not rare, we corrected odds ratio with Zhang's formula [20].

### Ethics statement

The Human Research Committee at the Kitasato University School of Medicine approved this study. We obtained a written consent with submitting a voluntary questionnaire.

## Results

We recruited 591 index patients and 1629 household contacts. Eighty-six patients did not return the questionnaire or respond to the interview and were excluded from the study. Table 1 shows the characteristics of index patients according to age group. The peak age band of index patients was 7–12 years old. Overall, 119 secondary cases occurred among 1629 household contacts, giving a SAR of 7.3% (95% CI: 6.1–8.7). Ninety-seven percent of index patients started antiviral medication within 48 hours of the onset of fever.

**Table 1:** Characteristics of the index patients according to age group.

	Total		0–6 Yr		7–12 Yr		13–19 Yr		20–29 Yr		30–39 Yr		≥40 Yr	
Category	(n = 591)		(n = 158)		(n = 232)		(n = 111)		(n = 32)		(n = 26)		(n = 32)	
Number of household contacts (including the index patient)														
Two to three	229	(39)	59	(37)	104	(45)	31	(28)	8	(25)	14	(54)	13	(41)
Four	283	(48)	82	(52)	101	(44)	59	(53)	16	(50)	9	(35)	16	(50)
Five or more	79	(13)	17	(11)	27	(11)	21	(19)	8	(25)	3	(11)	3	(9)
Number of secondary cases in household														
None	494	(84)	127	(80)	193	(83)	103	(93)	28	(88)	17	(65)	26	(81)
One	80	(14)	25	(16)	36	(16)	5	(5)	3	(9)	6	(23)	5	(16)
Two	12	(2)	6	(4)	1	(0)	1	(1)	1	(3)	2	(8)	1	(3)
Three	5	(1)	0	(0)	2	(1)	2	(2)	0	(0)	1	(4)	0	(0)
Treatment														
Zanamivir	296	(50)	22	(14)	149	(64)	103	(93)	7	(22)	6	(23)	9	(28)
Oseltamivir	295	(50)	136	(86)	83	(35)	8	(7)	25	(78)	20	(77)	23	(72)
Time interval from onset of fever to take the first medication														
Less than 48 hours	571	(96)	155	(98)	224	(97)	105	(95)	31	(97)	24	(92)	32	(100)
48 hours or more	20	(4)	3	(2)	8	(3)	6	(5)	1	(3)	2	(8)	0	(0)

CI; Confidence interval.

**Figure 1:**
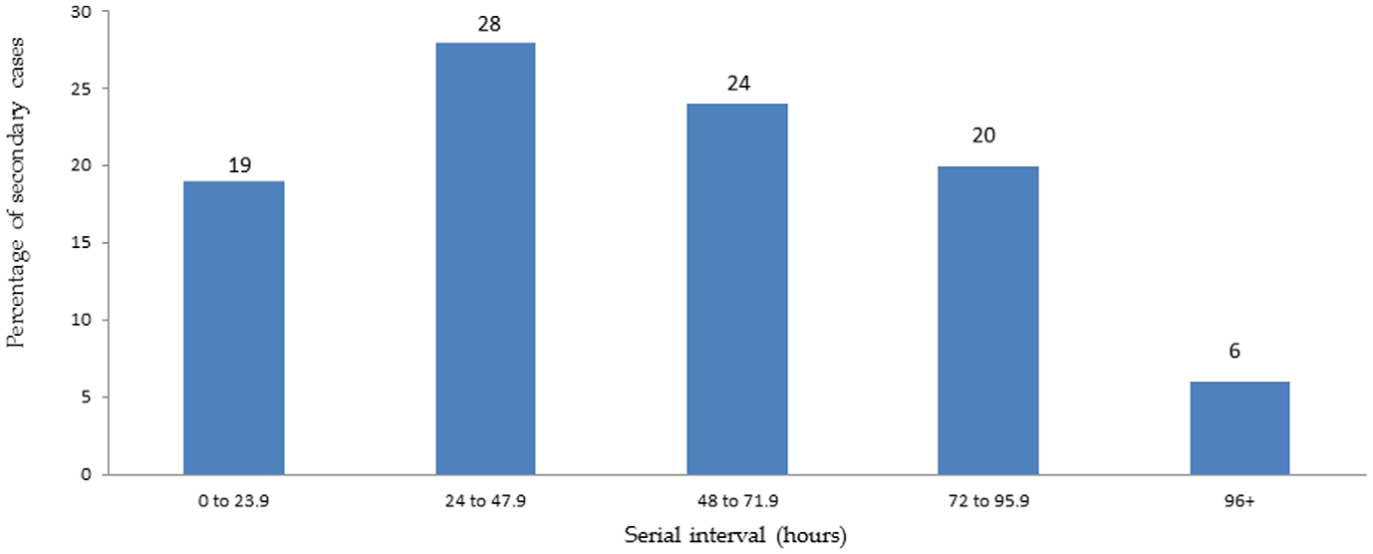
Distribution of days (serial interval) from onset of illness in the index patient to onset of influenza-like illness in the secondary case, Kawasaki, Japan 2009 (n = 97).

Figure 1 shows the distribution of days from onset of fever in the index patients to onset of influenza-like illness in the secondary cases. Half of the secondary cases developed fever within 48 hours after the index patient had fever. However, there were a few cases of household transmission at 96 hours after development of symptoms in the index patients.

Table 2 shows the association of household transmission with the study variables. The rate of household transmission by age of the index patient peaked among patients aged 30–39 years old with a SAR of 19.7 (11.8–31.0). For patients aged ≥30 years, only two index patients did not have any children, while the others with secondary transmission were mothers (22.9% of index mother patients) and fathers (36.8% of index father patients). The SAR were higher in households with more than five people (11.1 (7.7–14.5)) among patients who took oseltamivir (9.7 (7.7–11.8)); and when the time from onset of fever to initiating medication was 48 hours or more (18.3 (8.5–28.1)).

**Table 2:** Association between household transmission and the studied variables.

Category	With household transmission n = 97	Total n = 591	SAR (95% CI), %		*P*
Age of index patients, years					
0–6	31	158	8.8	(6.5–11.9)	<0.01
7–12	39	232	7.2	(5.5–9.6)	
13–19	8	111	3.3	(1.8–5.9)	
20–29	4	32	5.1	(1.9–11.6)	
30–39	9	26	19.7	(11.8–31.0)	
≥40	6	32	8.5	(3.9–16.9)	
Number of household contacts (including the index patient)					
Two to three	26	229	6.3	(4.0–8.5)	<0.01
Four	45	283	6.5	(4.8–8.2)	
Five or more	26	79	11.1	(7.7–14.5)	
Treatment					
Zanamivir	38	294	5.0	(3.5–6.6)	<0.01
Oseltamivir	59	293	9.7	(7.7–11.8)	
Time interval from onset of fever to taking the first medication					
Less than 48 hours	90	571	6.9	(5.7–8.2)	<0.01
48 hours or more	7	20	18.3	(8.5–28.1)	

CI: confidence interval.

SAR: secondary attack rate.

Table 3 shows logistic regression analysis between household transmission and the studied variables. Age of index patients [0–6 years old, odds ratio (OR): 2.56, 95% CI: 1.31–4.01; 7–12 years old, 2.44, 1.31–3.72; 30–39 years old 3.88, 2.09–5.21; ≥40 years old, 2.76, 1.17–4.53] and number of household members (five or more 3.09, 2.11–4.07) were significantly associated with household transmission. Differences in antiviral medication were significantly associated with household transmission in univariate analysis, however, it was not significantly associated in multivariate analysis. Initiation of antiviral medicine after 48 hours (2.38, 1.17–3.87) was significantly associated with household transmission.

**Table 3:** Risk factors for household transmission of pandemic H1N1 (2009) from index patients treated with antiviral medicine.

	Univariate analysis		Multivariate analysis	
Category	OR	(95%CI)	OR	(95%CI)
Age of index patients, years				
0–6	2.32	(1.30–3.56)	2.56	(1.31–4.01)
7–12	2.06	(1.14–3.24)	2.44	(1.31–3.72)
13–19	1		1	
20–29	1.62	(0.56–3.43)	1.44	(0.44–3.35)
30–39	3.49	(1.90–4.86)	3.88	(2.09–5.21)
≥40	2.24	(0.96–3.94)	2.76	(1.17–4.53)
Number of household contacts (including the index patient)				
Two to three	1		1	
Four	1.37	(0.90–2.00)	1.51	(0.98–2.19)
Five or more	2.62	(1.75–3.56)	3.09	(2.11–4.07)
Treatment				
Zanamivir	1		1	
Oseltamivir	1.55	(1.09–2.11)	1.20	(0.75–1.83)
Time interval from onset of fever to taking the first medication				
Less than 48 hours	1		1	
48 hours or more	2.19	(1.09–3.60)	2.38	(1.17–3.87)

## Discussion

The present prospective cohort study elucidated the risk factors that were associated with household transmission among the patients infected with influenza A, with antiviral drug treatment (oseltamivir or zanamivir) during the first wave of pandemic influenza virus (H1N1) 2009 at a primary clinic in Japan. We found that age ≤12 and ≥30 years old with children, households with more than five members, and initiation of antiviral therapy ≥48 hours were associated with household transmission. Differences in antiviral drug treatment were not significantly associated with household transmission based on logistic regression analysis, even though the SAR of zanamivir was significantly lower than that of oseltamivir.

The most identified type of influenza A virus from 22 July 2009 to 19 April 2010 according to national influenza surveillance in Japan was pandemic (H1N1) 2009 [21]. In the present study, we identified influenza A with a rapid antigen diagnostic test, and polymerase chain reaction testing was conducted by the Kawasaki City Institute for Public Health to confirm the pandemic (H1N1) 2009 virus for all 224 cases that were positive for influenza A until 21 October 2009. All the 242 cases were confirmed to be infected with pandemic (H1N1) 2009. We assumed that most patients were infected with pandemic (H1N1) 2009 virus in the study period. Vaccination against pandemic (H1N1) 2009 virus in Japan had been provided according to the list of prioritized groups from November 2009 and became available for everyone from January 2010. Thus, we did not take the effect of vaccination into account in this study.

The age group that was most frequently infected by pandemic (H1N1) 2009 virus in Japan in 2009 was 10–19 years old, and 65% of this age group were infected in the first wave of 2009, according to a national survey of antibody titer [22]. In our study, the rate of secondary transmission among the index patients who were 13–19 years old (3.3%) and 20–29 years old (5.1%) was lower compared with other age groups. It is possible that those aged 13–29 years maintained a distance from other household contacts to avoid secondary transmission at home. It has been shown previously that there is a high risk of household transmission when index patients are preschool age [4], [6], [23]–[25], whereas we found that people aged ≥30 years also had a high risk of household transmission, as well as those who were ≤12 years old. All of those who were ≥30 years of age in the present study were parents, and it is possible that they might not avoid close contact when taking care of their children at home. Children aged ≤12 years old tend to have longer periods of shedding influenza virus and do not comply with hygiene and precautions against infection [4], [25]–[27].

Our study showed that a larger household with more than five members or more was a risk factor even though some studies indicated household size has not been determined as a risk factor for domestic transmission [4], [22]. Family size and the number of children are recognized as risk factors for household transmission in previous studies [5], [28], [29]. However, contact between patients and healthy persons in the household could differ based on cultural and familial factors and the size of the house [24].

The effectiveness of antiviral medication (zanamivir or oseltamivir) to reduce household transmission in index patients is controversial [16], [30]–[32]. Antiviral medication for patients could have some effect on preventing household transmission. Nishiura et al. have shown that zanamivir reduces the risk of household transmission among patients who are infected with pandemic (H1N1) 2009 virus in Japan [33]. In our study, there was a significant difference of the SAR between zanamivir and oseltamivir for the index patients with regard to household transmission; however the logistic regression analysis did not identify a significant difference. This was possibly due to the limited number of participants, which influenced our ability to detect the difference. Based on the age stratified analysis, the population treated with zanamivir had a lower risk of secondary transmission even though there was a bias for prescribing zanamivir for 10–19 year-old patients who had lower secondary attack rate resulting in risk of indicating a spurious preventing effect. Further studies are needed to elucidate the effectiveness of each antiviral medication for prevention of household transmission.

Early antiviral medication might minimize the risk of household transmission. Ng et al. have shown that treatment by oseltamivir within 24 hours reduced household transmission of influenza significantly compared with no antiviral treatment [16]. Pebody et al. have shown a significant reduction of influenza with antiviral treatment of the index patient within 48 hours [32]. Our study also showed that antiviral treatment after 48 hours resulted in a higher risk of household transmission, when compared with a group with early antiviral treatment, although there were a limited number of patients who had antiviral treatment after 48 hours. However, the interval before secondary transmission indicates how quickly an epidemic can evolve in a household [2], [6]. Half of patients with household transmission of influenza have symptoms within 48 hours of symptom onset in index patients. Transmission occurs very early in the development of symptoms in the index patient, which might not be preventable by early antiviral treatment because of the time required to obtain antiviral medicine at a clinic. In addition, shedding of seasonal influenza virus has been identified before one day of peak of influenza like symptoms [34]. Further studies are needed to elucidate how much early treatment could be effective for preventing household transmission, and how early people should take antiviral medication for preventing household transmission. Physical intervention for preventing household transmission is also needed, such as hand washing, especially in the early phase of illness. However, measures such as maintaining distance from or isolation of the patient is difficult if small children are infected [35], [36].

Some of the secondary household transmission cases in this study might have been caused by infections from outside of their household, as many people were infected with pandemic (H1N1) 2009 in their communities during the study period. However, the partial correlation coefficients between the weekly accumulated secondary transmission cases, and the weekly-accumulated number of influenza patients who did not have any family members diagnosed with influenza within seven days, which could reflect the community transmission in this data setting was −0.00019. This was adjusted for the weekly-accumulated numbers of first patients in their households. Thus, the number of patients infected with pandemic (H1N1) 2009 outside of their household was likely to be minimal, although further studies are still needed to elucidate the impact of these cases on secondary household transmission of influenza with mathematical modeling.

There were a few limitations in our study. First, the generalizability of our results was limited to the population treated with antiviral medicine. Second, we neither followed-up patients by home visits nor disregarded the possible significance of subclinical or asymptomatic infection. Third, biases could have further arisen from the potential that all index patients recruited had to be sick enough to initially seek medical attention, thus selecting for “sicker” patients who may have had a higher degree of “infectiousness”.

Household transmission was associated with index patients aged <12 years old and adults ≥30 years with children; more than five persons in the household; initiation of treatment at ≥48 hours from onset of fever within the population treated with antiviral medicine. We need to warn patients at high risk of household transmission to take additional precautions.

## References

[1] Papenburg J, Baz M, Hamelin ME, Rheaume C, Carbonneau J (2010). Household transmission of the 2009 pandemic A/H1N1 influenza virus: elevated laboratory-confirmed secondary attack rates and evidence of asymptomatic infections.. Clin Infect Dis.

[2] Suess T, Buchholz U, Dupke S, Grunow R, an der Heiden M (2010). Shedding and transmission of novel influenza virus A/H1N1 infection in households–Germany, 2009.. Am J Epidemiol.

[3] Simmerman JM, Suntarattiwong P, Levy J, Gibbons RV, Cruz C (2010). Influenza virus contamination of common household surfaces during the 2009 influenza A (H1N1) pandemic in Bangkok, Thailand: implications for contact transmission.. Clin Infect Dis.

[4] Carcione D, Giele CM, Goggin LS, Kwan KS, Smith DW (2011). Secondary attack rate of pandemic influenza A(H1N1) 2009 in Western Australian households, 29 May–7 August 2009.. Euro Surveill.

[5] Savage R, Whelan M, Johnson I, Rea E, Lafreniere M (2011). Assessing secondary attack rates among household contacts at the beginning of the influenza A (H1N1) pandemic in Ontario, Canada, April–June 2009: A prospective, observational study.. BMC Public Health.

[6] France AM, Jackson M, Schrag S, Lynch M, Zimmerman C (2010). Household transmission of 2009 influenza A (H1N1) virus after a school-based outbreak in New York City, April–May 2009.. J Infect Dis.

[7] Yang Y, van Boven M, Donker T, van der Lubben M, van Gageldonk-Lafeber RB (2010). Transmission of Novel Influenza A(H1N1) in Households with Post-Exposure Antiviral Prophylaxis.. PLoS One.

[8] Meltzer MI, Cox NJ, Fukuda K (1999). The economic impact of pandemic influenza in the United States: priorities for intervention.. Emerg Infect Dis.

[9] Carrat F, Sahler C, Rogez S, Leruez-Ville M, Freymuth F (2002). Influenza burden of illness: estimates from a national prospective survey of household contacts in France.. Arch Intern Med.

[10] Cowling BJ, Chan KH, Fang VJ, Lau LL, So HC (2010). Comparative epidemiology of pandemic and seasonal influenza A in households.. N Engl J Med.

[11] Yang Y, Sugimoto JD, Halloran ME, Basta NE, Chao DL (2009). The transmissibility and control of pandemic influenza A (H1N1) virus.. Science.

[12] Kamigaki T, Oshitani H (2009). Epidemiological characteristics and low case fatality rate of pandemic (H1N1) 2009 in Japan.. PLoS Curr.

[13] Wada K, Nishiura H, Kawana A (2010). An epidemiological analysis of severe cases of the influenza A (H1N1) 2009 virus infection in Japan.. Influenza Other Respi Viruses.

[14] Ikeda N, Saito E, Kondo N, Inoue M, Ikeda S (2011). What has made the population of Japan healthy?. The Lancet.

[15] Hayward A (2010). Does treatment with oseltamivir prevent transmission of influenza to household contacts?. Clin Infect Dis.

[16] Ng S, Cowling BJ, Fang VJ, Chan KH, Ip DK (2010). Effects of oseltamivir treatment on duration of clinical illness and viral shedding and household transmission of influenza virus.. Clin Infect Dis.

[17] Moscona A (2005). Neuraminidase inhibitors for influenza.. N Engl J Med.

[18] Center for Disease Control and Prevention (2009). Outbreak of swine-origin influenza A (H1N1) virus infection - Mexico, March–April 2009.. MMWR Morb Mortal Wkly Rep.

[19] Hama R (2007). Oseltamivir's adverse reactions: fifty sudden deaths may be related to central suppression.. BMJ.

[20] Zhang JYK (1998). What's the relative risk? a method of correcting the odds ratio in cohort studies of common outcomes.. JAMA.

[21] National Institute of Infectious Diseaes, Japan (2009). Current situation of pandemic influenza A (H1N1).. http://idsc.nih.go.jp/disease/swine_influenza/2009idsc/09idsc29.html.

[22] National Institute of Infectious Diseaes, Japan (2009). Report of a survey on antibody of Influenza A/California /7/2009pdm.. http://idsc.nih.go.jp/yosoku/Flu/2010Flu/Flu10_1.html.

[23] Komiya N, Gu Y, Kamiya H, Yahata Y, Yasui Y (2010). Household transmission of pandemic 2009 influenza A (H1N1) virus in Osaka, Japan in May 2009.. J Infect.

[24] Cauchemez S, Donnelly CA, Reed C, Ghani AC, Fraser C (2009). Household transmission of 2009 pandemic influenza A (H1N1) virus in the United States.. N Engl J Med.

[25] Hirotsu N, Iwaki N, Kawai N, Ikematsu H, Kashiwagi S, Kats JM (2007). Intra-Familial Transmission of Influenza A and Influenza B..

[26] Longini IM, Koopman JS, Monto AS, Fox JP (1982). Estimating household and community transmission parameters for influenza.. Am J Epidemiol.

[27] Ling LM, Chow AL, Lye DC, Tan AS, Krishnan P (2010). Effects of early oseltamivir therapy on viral shedding in 2009 pandemic influenza A (H1N1) virus infection.. Clin Infect Dis.

[28] Goldstein E, Cowling BJ, O'Hagan JJ, Danon L, Fang VJ (2010). Oseltamivir for treatment and prevention of pandemic influenza A/H1N1 virus infection in households, Milwaukee, 2009.. BMC Infect Dis.

[29] Loustalot F, Silk BJ, Gaither A, Shim T, Lamias M (2011). Household transmission of 2009 pandemic influenza A (H1N1) and nonpharmaceutical interventions among households of high school students in San Antonio, Texas.. Clin Infect Dis.

[30] Halloran ME, Hayden FG, Yang Y, Longini IM, Monto AS (2007). Antiviral effects on influenza viral transmission and pathogenicity: observations from household-based trials.. Am J Epidemiol.

[31] Looker C, Carville K, Grant K, Kelly H (2010). Influenza A (H1N1) in Victoria, Australia: a community case series and analysis of household transmission.. PLoS One.

[32] Pebody RG, Harris R, Kafatos G, Chamberland M, Campbell C (2011). Use of Antiviral Drugs to Reduce Household Transmission of Pandemic (H1N1) 2009, United Kingdom.. Emerg Infect Dis.

[33] Nishiura H, Oshitani H (2011). Household Transmission of Influenza (H1N1-2009) in Japan: Age-specificity and Reduction of Household Transmission Risk by Zanamivir Treatment.. J Int Med Res.

[34] Carrat F, Vergu E, Ferguson NM, Lemaitre M, Cauchemez S (2008). Time lines of infection and disease in human influenza: a review of volunteer challenge studies.. Am J Epidemiol.

[35] Cowling BJ, Chan KH, Fang VJ, Cheng CK, Fung RO (2009). Facemasks and hand hygiene to prevent influenza transmission in households: a cluster randomized trial.. Ann Intern Med.

[36] Jefferson T, Del Mar CB, Dooley L, Ferroni E, Al-Ansary LA (2011). Physical interventions to interrupt or reduce the spread of respiratory viruses.. Cochrane database of systematic reviews.

